# A Conversation with David Sullivan, MD, Professor, Molecular Microbiology and Immunology, Johns Hopkins Bloomberg School of Public Health

**DOI:** 10.1017/cts.2023.663

**Published:** 2023-12-11

**Authors:** Clinical Research Forum

**Affiliations:** Clinical Research Forum, Washington, DC, USA

## Top 10 Clinical Research Achievement Awards Q & A

This article is part of a series of interviews with recipients of Clinical Research Forum’s Top 10 Clinical Research Achievement Awards. This article is with Dr David Sullivan, MD, Professor, Molecular Microbiology and Immunology, Johns Hopkins Bloomberg School of Public Health. Dr Sullivan specializes in infectious disease and received a 2023 Top 10 Clinical Research Achievement Award for *Early Outpatient Treatment for Covid-19 with Convalescent Plasma. The interview has been edited for length and clarity.*

## You’re an infectious disease doctor who is focused on developing novel malaria diagnostics and drugs. What first got you interested in malaria?

I’d flip the question. I don't understand why everyone else is *not* interested in malaria. Historically, it has been underrepresented in research among single infectious disease killers even though more than 600,000 people die from malaria each year, most of them under the age of five. Ever since I was a medical resident, I knew I wanted to work in this field, and I’ve been working principally at the bench on malaria diagnostics and drugs.

## But in the spring of 2020 your research priorities shifted?

Yes. When the pandemic happened, I totally pivoted from the benchwork I was doing and turned to COVID-19 clinical research. Johns Hopkins is a great place for combining different research elements and back then it was “all-hands-on-deck.” Because I was working on diagnostics and drugs, I was able to bring that mindset to antibody therapy, especially with regard to the general principles of early treatment with high doses. The award-winning research was actually my first clinical trial, and I feel fortunate that I had the right talents at the right time in the right environment.

## It must have been challenging running your first clinical trial during the pandemic

Yes and no. Most clinical researchers take a year or two to think of an idea, get the funding right, and then get their trial started. But we were able to do all that in a few months. We launched with a few million in seed money, and then a colleague I knew through my work on malaria connected me with someone in the Department of Defense. Because passive antibody therapy was within their realm under Operation Warp Speed, we received more funding and the project ramped up quickly from there. Still, there was a lot of behind-the-scenes work, getting things lined up, and with mountains to move, we did whatever it took. For example, when our trial began most hospitals and outpatient centers weren't allowing infected patients in the building, so for many of our sites, we used pods in parking lots. We had to adapt and work with electricians and engineers to put everything together. It was like figuring out an intricate multifaceted puzzle and I took whatever lessons I learned at one site and translated those to the others. In some respects, it was probably a good thing that this was my first trial. I didn't have any preconceived notions and everything was on the table.

## What were your main takeaways on a personal level?

I learned that running a clinical trial requires patience, collaboration, and hope. I developed the catch phrase “pleasant, relentless persistence”—because that’s what is needed, across all aspects, starting from the very beginning, with funding. I also want to stress that it really was a team approach and everyone here rose to action, so to speak. I stood on the shoulders of Dan Hanley, Dan Ford, and all those at the Johns Hopkins Clinical Research Network. We shared our expertise and gave encouragement to one another at weekly meetings. We all wanted to be part of the solution—and that includes the participants in trial. In many respects, convalescent plasma is “the people’s plasma.” It’s not from a drug company or an academic center. It’s donated by people. I think all of us were inspired by one another and knew we were contributing to something bigger.

## What are the public health implications of this trial?

Our trial showed that plasma containing viral antibodies from the blood of recovered COVID-19 patients safely reduced hospitalization risk by 54% in early outpatient treatment. That changed clinical practice—within a week of the published results, the FDA authorized outpatient convalescent plasma currently benefiting the immunocompromised. The research we did provides a blueprint of hope for early outpatient high antibody dose convalescent plasma for rapid treatment response for future pandemics while monoclonal therapies and vaccines are being developed. This can be especially important in resource-constrained areas or where there are imbalances in vaccine distribution. In addition, we are continuing to work with the Association for the Advancement of Blood and Biotherapies (AABB) and other agencies to better understand the appropriate use and efficacy of COVID-19 convalescent plasma for those immunocompromised not generating antibodies on their own.

## Do you see yourself doing more clinical studies in the future?

Yes, I do. Running a clinical trial is a complex exercise. You’re constantly balancing people—patients, clinicians, others—all making their own individual decisions. But looking back, it was a fun challenge to orchestrate and take it all the way to publication. For now, I'm back to bench research. There is a lot of follow-up based on information we collected and bench research we started during the trial. We need to follow through so that we can take what we learn and apply it to future pandemics. Again, it comes down to “pleasant, relentless persistence.”

## Does that come naturally to you?

Lots of people are hard workers during a pandemic, so I'm no different than anyone else in that respect. But I do have an engineering mindset and I like sticking with a challenge. Outside of the lab I like to hike and I’ve also been a long distance runner and an avid biker. I used to bike to work all year long, in the rain, in the snow—so yes, I guess there are clues there about my persistence. But it’s also really motivating, whether you’re doing bench work or a clinical trial, to know that what you’re doing is going to help people. That’s where I draw the most inspiration—patients.

